# Middle Cerebral Artery Territory Ischemic Infarction Secondary to Russell’s Viper Envenomation: A Case Report

**DOI:** 10.7759/cureus.96290

**Published:** 2025-11-07

**Authors:** Vijayakumaran Yanusha, Pakkiyaretnam Mayurathan, Sithy Sabrina

**Affiliations:** 1 Internal Medicine, Batticaloa Teaching Hospital, Batticaloa, LKA; 2 University Medical Unit, Batticaloa Teaching Hospital, Batticaloa, LKA; 3 Clinical Sciences, Faculty of Health-Care Sciences, Eastern University of Sri Lanka, Batticaloa, LKA; 4 Medicine, Batticaloa Teaching Hospital, Batticaloa, LKA

**Keywords:** hemotoxic, ischemic stroke, russell's viper envenomation, vicc, wbct20

## Abstract

In tropical countries such as Sri Lanka, snake envenomation represents a significant public health concern. Russell’s viper is one of the venomous snakes, causing local and systemic manifestations. Ischemic stroke following Russell’s viper bite is rare and is underreported. We describe a case of a 40-year-old previously healthy man who developed a left middle cerebral artery territory infarction following a Russell’s viper bite, despite the timely administration of snake antivenom. Non-contrast computed tomography (NCCT) brain revealed left-sided middle cerebral artery territory ischemic infarction. Evaluation for stroke excluded common aetiologies, including atherosclerosis, cardiac embolism, and primary vasculitis. This suggests that the most plausible mechanism for the infarction is toxin-induced vasculitis and endothelial injury.

## Introduction

Worldwide, snakebite is a major yet underrecognized public health challenge, affecting an estimated 5.4 million people annually, with 1.8 to 2.7 million cases of envenoming [1]. In Sri Lanka, envenoming due to Russell’s viper (Daboia russelii) has been documented across all climatic zones [2]. It causes local and systemic clinical manifestations, including bite site swelling, necrosis, neurotoxicity, nephrotoxicity, and coagulopathy [2,3]. The most common neurological manifestations are cranial nerve involvement, typically presenting as ptosis and ophthalmoplegia [2,3,4]. Cerebrovascular accidents are rare complications of Russell’s viper bite, with intracranial haemorrhages occurring more frequently than infarctions [5].

## Case presentation

**Figure 1:**
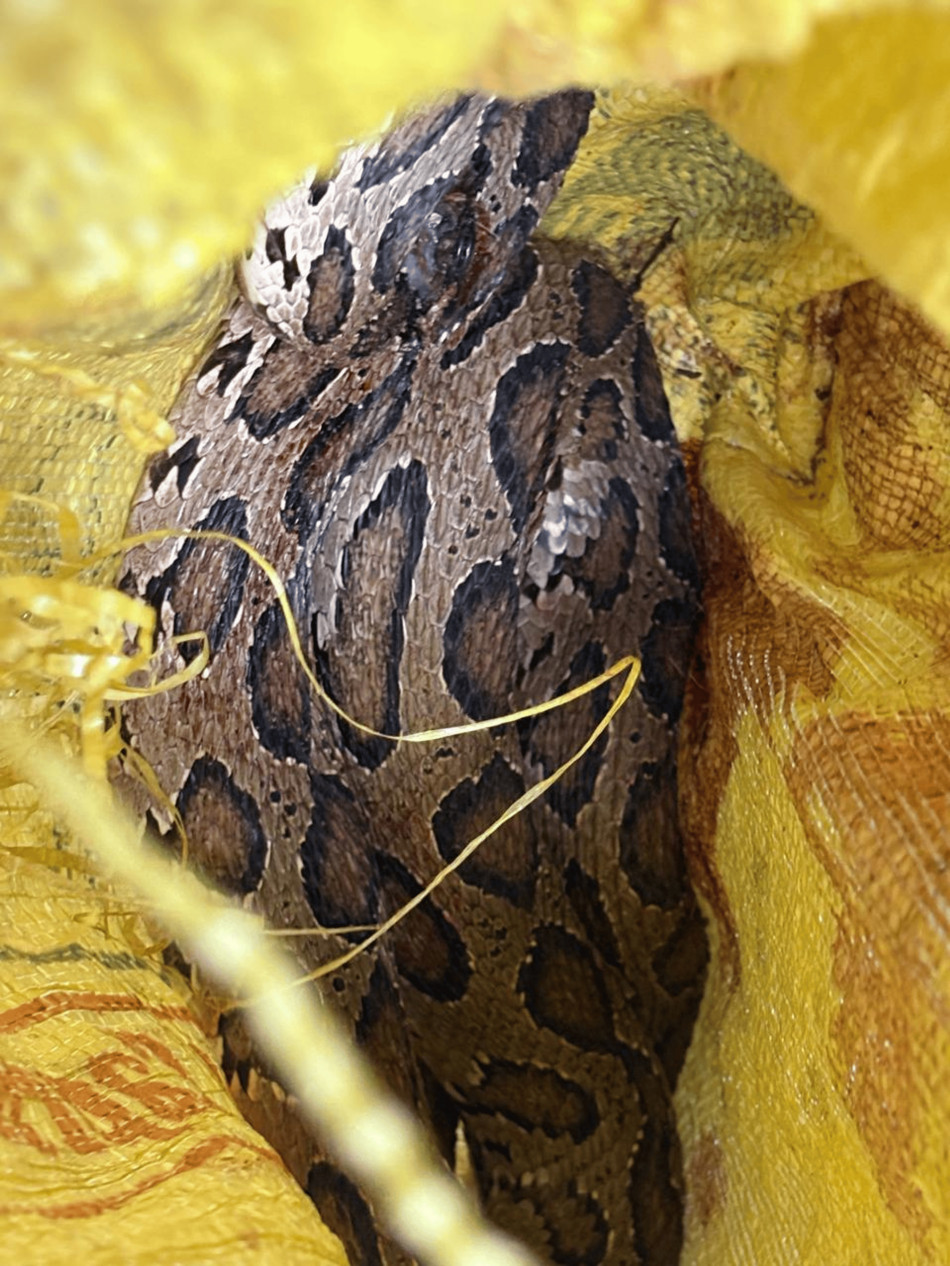
Russell's viper Image credit: author's original creation

A 40-year-old previously unevaluated gentleman was admitted to the local hospital (LH) with a snake bite on the left foot two hours back. On admission, he had bilateral ptosis with swelling of the bite area. He had a prolonged 20-minute whole blood clotting test (WBCT20) immediately, with 20 vials of snake antivenom given. The snake (Figure 1) had been identified as Russell’s viper (D*aboia russelli*). After six hours of admission, WBC 20 became normal. 

**Figure 2:**
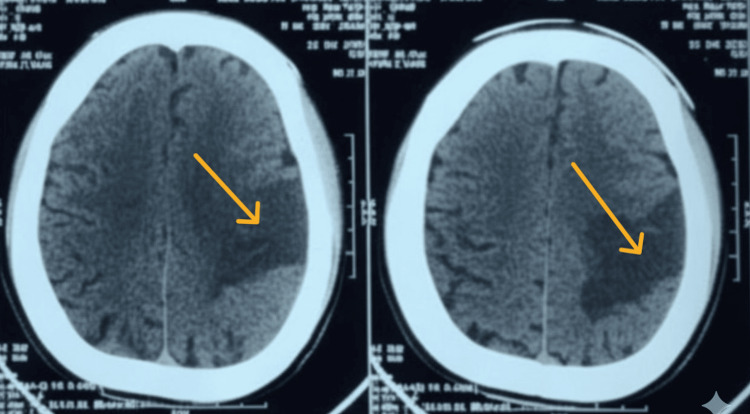
Left-sided middle cerebral artery territory infarction

The next day, while waking up from sleep, he noticed right-sided upper limb and lower limb weakness. He was immediately transferred to the Batticaloa Teaching Hospital (THB) for non-contrast computed tomography (NCCT) brain and further management. On admission to THB, he complained of right-sided weakness and left lower limb pain and swelling. His Glasgow Coma Scale (GCS) score was 15/15 (E4V5M6). On examination, a left foot fang mark with edema was noted. His blood pressure was 110/78 mmHg, pulse rate was 94/min, SpO2 was 98% on room air, and respiratory rate was 18/min. Neurological examination showed right-sided upper and lower limb power 1/5, pupils were bilaterally equal with 3 mm reactive to light, and slurred speech with left-sided mouth deviation. NCCT-brain (Figure 2) showed an acute infarct in the left middle cerebral artery territory. He was initially treated with intravenous co-amoxiclav, aspirin, and atorvastatin. We evaluated for other causes of ischemic stroke. Clotting profile was normal, and 2D echocardiography showed a normal study with an ejection fraction of 60% without valvular pathology or intracardiac thrombus. Carotid and vertebral artery Doppler studies were unremarkable.

On the following day, he became agitated and confused. He developed respiratory distress and was intubated and transferred to the intensive care unit (ICU). ECG was normal, and other investigations are given below. Chest X-ray shows bilateral perihilar patchy consolidation suggestive of pulmonary edema.

During ICU care, his renal function worsened dramatically. He was managed with an infusion. His hemoglobin gradually dropped without any obvious bleeding manifestations. His blood picture did not show evidence of microangiopathic hemolytic anemia. But LDH was slightly elevated with normal retic count.

On the 16th day of ICU care, his urine output became less than 10 ml for six hours, and respiratory distress worsened. Hemodialysis was started, and multiple blood transfusions were given. With the gradual improvement of sensorium, he was extubated after 20 days of ICU care. His investigations demonstrated improvement during admission (Table 1). A structured rehabilitation program was initiated, including daily physiotherapy and speech therapy. The patient demonstrated slow but steady functional improvement and was subsequently transferred to the LH for ongoing care.

**Table 1:** Investigations during admission LH: local hospital; WBCT: whole blood clotting time; INR: international normalized ratio; aPTT: activated partial thromboplastin time; AST: aspartate transaminase; ALT: alanine transaminase; LDH: lactate dehydrogenase; WBC: white blood cell

Investigation	Normal range	On admission to LH	On admission to TCH	On admission to ICU	3^rd^ day of ICU	5^th ^day of ICU	16^th^ day of ICU	20^th^ day of ICU	Before transfer to LH
WBCT 20min	<20 min	Prolonged	<20 min	<20 min	-	-	-	-	-
Hemoglobin	12-16 g/dl	13	13	13	10	9	8.2	9.5	9.4
WBC	4-11 x10^3^/uL	12.4	13.69	10.7	15.9	16.37	12.2	14.2	12.7
Platelet	150-450 x10^3^/uL	100	58	122	172	193	209	163	185
Retic count	-	-	0.46%	-	-	-	-	-	-
LDH	-	-	480	-	470	-	-	-	-
Serum creatinine	62-115 µmol/L	78	654	872	706	571	520	320	280
CRP	0-5mg/L	-	50	208	130	68	33	18	13
Sodium	136-145 mmol/L	-	131	133	155	156	149	135	-
Potassium	3.5-5.1 mmol/L	-	3.7	4.7	4.3	4.1	3.9	4.8	-
ALT	12-78 IU/L	-	40	30	-	-	110	78	-
AST	15-37 IU/L	-	26	14	-	-	104	56	-
INR	-	2.1	1.04	1.26	-	1.48	1.2	1.2	1.04
aPTT	Sec	76	33.5	26.9	-	-	-	-	30
Urine full report	-	-	Albumin+++; pus cell-field full/hpf; RBC-Nil dysmorphic RBC-Nil	-	-	-	-	-	Albumin++; pus cell-2-3/hpf; RBC-Nil
Creatine phosphokinase(U/L)	-	-	716		370	-	-	-	-

## Discussion

Russell’s viper envenomation causes local and systemic clinical manifestations, including local bite site swelling, necrosis, neurotoxicity, nephrotoxicity, venom-induced coagulopathy, and bleeding diathesis [2,3]. A large Sri Lankan case series of 336 patients with confirmed Russell's viper envenomation revealed neurological involvement in approximately 78% of cases [6]. Ptosis and ophthalmoplegia are the mostly observed neurological manifestations [2-4,6]. Only a few cases have been reported as ischemic cerebral infarction [5,6]. Snake venom can cause a stroke due to either their neurotoxic or hemotoxic enzymes. Depending on the enzyme makeup type, it may vary from hemorrhagic or ischemic [7].

The pathophysiological mechanisms underlying cerebral infarction following Russell’s viper bite are multifactorial and remain incompletely elucidated, with potential contributors including venom-induced procoagulant activity leading to microthrombi formation, complement-mediated vasculitis, direct endothelial injury, cardiotoxic effects resulting in arrhythmias and embolism, and hypovolemia-associated hyper viscosity [7]. Phospholipase A₂ and other toxic venom components may further contribute to vascular dysfunction [7,8]. In this case, ischemic infarction due to hypotension is unlikely, as he remained normotensive and no watershed infarcts were observed on CT brain imaging. The lipid profile was normal, and there was no history of smoking. A cardiac source of embolization was also excluded. His blood picture showed no evidence of thrombotic microangiopathy. The absence of conventional stroke risk factors such as dyslipidemia, smoking, arrhythmias, hypotension, or cardiac emboli, together with the presence of ischemic infarct, strongly supports a systemic toxin-mediated mechanism, likely involving toxin-induced vasculitis with procoagulant activity and platelet aggregation.

Our patient developed respiratory distress, one of the common features of severe systemic envenomation. It may result from pulmonary edema, neurotoxic respiratory muscle paralysis, or severe metabolic derangements [8]. In this case, a chest X-ray shows evidence of pulmonary edema, and other investigations show metabolic derangements. It was most likely due to a combination of all these factors.

Acute kidney injury (AKI) is a recognized complication of snake envenoming, with emerging evidence indicating that it is a major contributor to snakebite-associated morbidity and mortality, and severe cases often necessitate hemodialysis. The pathophysiology remains poorly understood, with limited evidence supporting the presence of primary or direct nephrotoxins in snake venom. Many other mechanisms have been suggested, including AKI secondary to hypotension, venom-induced consumption coagulopathy, rhabdomyolysis, and microangiopathic hemolytic anemia [9]. In our case, the patient developed severe AKI following Russell’s viper envenoming, which required multiple sessions of hemodialysis for management. The AKI in our case is possibly due to multifactorial such as venom-induced consumption coagulopathy and rhabdomyolysis.

Russell’s viper envenomation can lead to hemoglobin loss because of venom-induced consumption coagulopathy (VICC), microangiopathic hemolytic anemia, AKI, and bleeding manifestations secondary to coagulopathy [2,3]. In our case, we observed a rapid decline in hemoglobin levels. As the peripheral blood smear did not indicate hemolysis, the fall is most likely attributable to venom-induced coagulopathy and concurrent AKI.

## Conclusions

Ischemic stroke following Russell’s viper envenomation is a rare complication but is associated with significant morbidity and mortality. Even with timely antivenom therapy, certain systemic manifestations may remain unavoidable. Therefore, prevention of snakebite is ultimately more effective than managing its complications.
